# An Analysis of the Effects of Psychosocial Work Environment on the Retention Intentions of Primary Care Coordinators Nursing Patients with Chronic Illness

**DOI:** 10.3390/ijerph182212104

**Published:** 2021-11-18

**Authors:** Hyesoo Lee, Juh Hyun Shin

**Affiliations:** College of Nursing, Ewha Womans University, Seoul 03760, Korea; juhshin@ewha.ac.kr

**Keywords:** nurses, care coordinators, chronic illness management, psychosocial work environment, intent to stay

## Abstract

This study aimed to investigate the effects of psychosocial work environment on the retention intentions of care coordinators taking care of patients with chronic illness. A descriptive survey study was conducted with a convenience sample of care coordinators who organized patients and treatment teams that offered professional and persistent treatment. A total of 132 participants were recruited from 19 October to 19 November 2020. The data were analyzed through descriptive statistics, t-tests, ANOVA, Scheffé post hoc, and hierarchical multiple regression using SPSS 26.0. The results showed that work organization and job content (β = 0.254, *p* = 0.014) and value at the workplace (β = 0.245, *p* = 0.034) had significant effects on the retention intentions of participants. The final model of the study explained 40.1% of participants’ retention intentions (F = 11.830, *p* < 0.001). The development of educational programs and implementation of policies for improving the psychosocial work environment were found to be essential for increasing the retention intentions of professional care coordinators.

## 1. Introduction

Chronic diseases are defined as diseases that continuously affect patients’ daily lives and require periodic management for at least a year [1]. About 17.3 million people with chronic diseases reside in Korea, which constitutes 33.6% of the population [2]. Furthermore, 79.8% of all deaths were caused by chronic diseases [3]. Additionally, coronavirus disease 2019 (COVID-19) broke out in 2019 and spread rapidly throughout the world. The disease causes complications in patients with chronic diseases such as cardiovascular diseases, hypertension, chronic respiratory disease, metabolic syndrome, and diabetes, often leading to critical and fatal illness [4]. The Chronic Care Model (CCM) to reinforce primary care has been proposed as a solution to chronic disease treatment worldwide [5].

Since the Korean Liberation in 1945, public healthcare infrastructure has barely been developed. Instead, private sectors take care of patients with chronic disease without formal regulation or any central supervising organization [6]. Chronic disease management mainly depends on the private sector and public health clinics’ segmented and dualized structure. To offset this problem, the Korean government has made various attempts to manage chronic diseases and reduce medical care costs, such as “Korean Hypertension Diabetes Daegu Initiative (2007)”, “Community-based Primary Care Project (2014)”, and “Information and Communication Technologies based Health Care Management Project (2016)”. However, the results have not been significant [2].

Healthcare clinics are settings that provide healthcare services mostly to outpatients and are suitable for managing chronic diseases that require regular visits and prescriptions [7]. Integrated health education and counseling are important for the management of chronic diseases, and nurses play a key role in team approaches as a continuous, integrated, and patient-centered model is required, rather than a segmental model [8]. Although professional nurses ensure the quality and continuity of nursing care and health-care services for patients, which are essential in managing chronic disease [9], nurses are not actively employed to continuously monitor chronic disease patients at clinic-level healthcare institutions in Korea. As of 2020, only 7.4% of nurses in the country were estimated to be working in clinics [10]. As of 2016, 75.2% of clinics had no nurses, and fewer than 10% did not replace nurses with nursing assistants [11]. Additionally, the lack of incentives to retain working nurses led to a decrease in the proportion of nurses in clinics in 2020 compared to that in 2018 [10], before the introduction of the “Primary Health Care Chronic Diseases Management Pilot Program”.

In 2019, the South Korean Ministry of Health and Welfare introduced the “Primary Health Care Chronic Diseases Management Pilot Program” to effectively manage patients with chronic diseases. This was the first project to encourage hiring care coordinators who are certified dietitians or registered nurses. The project establishes a foundation for the active and effective management of patients with chronic diseases in clinic-level healthcare institutions with a framework of care coordinators who monitor patient management in primary care, provide patient education and counseling, and connect patients to local communities and tertiary hospitals [12]. The care coordinators coordinate patients, caregivers, doctors, and communities to manage chronic diseases [2]. However, coordinators focus on coordinating and sharing information on the changes in patient health with team members and community health providers [13].

To ensure skilled nurses perform their roles properly, various studies have explored factors that influence nurses’ retention. In a United Kingdom-based study involving nurses working in primary care settings and local communities, the retention intentions of nurses were found to be higher when managers and patients respected nurses and their autonomy at work [14]. In another Korean study of nurses in small and medium-sized hospitals, the work environment was identified as a key factor affecting retention intentions [15]. Nurses’ psychosocial work environment refers to their overall working environment, including physical and human environments, that enable nurses to perform their tasks efficiently. Improving nurses’ work environment has been suggested as a measure to improve nurse shortages and turnovers [10]. Poor work environment is an impeding factor for high-quality nursing performance, leading to negative effects on nurses’ retention and decreased quality of nursing care and patient satisfaction [16]. Based on these factors, the impact of the social and psychological work environment, indicated by relationships, personal work, and workplace culture, and the physical environment is increasing [17]. The psychosocial work environment is a key field of research to understand psycho-physiological processes and health and illness behaviors while linking economics, society, politics and disease [18].

Currently, nurses play varied and complex roles between healthcare institutions and patients. The duties of nurses have expanded from direct nursing to interacting with other staff and considering the economic aspects of organizations and institutions [19]. In particular, care coordinators cooperate with doctors and patients, share treatment progress, and collaborate with healthcare institutions as well as public and private resources in local communities for patient management [2]. Therefore, the work environment of nurses must be improved by promoting a cooperative culture, establishing open communication, including nurses in decision making, demarcating professional boundaries, and providing adequate compensation for high-quality nursing care [20]. Furthermore, care coordinators must establish care plans through comprehensive evaluations of patients with chronic disease in addition to performing the tasks assigned to nurses, such as providing education on drug use and lifestyle habits, preventing complications according to care plans, and functioning as a link between local communities and tertiary hospitals [2]. It is important to recognize that nurses’ psychological environment affects their cognition, emotions, behaviors, and psychophysiology. Therefore, studies must simultaneously investigate nurses’ psychological and social environments, including social structures and interactions between individuals and groups. Extensive and comprehensive assessments of nurses’ psychosocial work environment can contribute towards the improvement of these work environment [21], while also reducing problems due to turnover and absences by increasing work satisfaction [20]. Such assessments are ideal for studies undertaken with the purpose of identifying potential measures to increase the retention intentions of care coordinators.

Therefore, this study evaluated the effects of the psychosocial work environment on the retention intentions of care coordinators. Ultimately, the study aimed to provide basic data for the safe and high-quality nursing of patients with chronic illness in primary care.

## 2. Materials and Methods

### 2.1. Study Design

This was a descriptive research study to understand the psychosocial work environment and retention intentions of chronic disease management care coordinators and analyze the effects of the psychosocial work environment on retention intentions.

### 2.2. Participants

This study involved nurses working as care coordinators at clinic-level healthcare institutions registered for the “Primary Health Care Chronic Diseases Management Pilot Program.” Nurses who were not working as care coordinators or who worked in clinics without patients participating in the pilot program were excluded. A total of 165 care coordinators from 120 institutions were recruited, and a total of 132 questionnaires were collected and included in the final analysis. This number satisfied the minimum sample size of 114, calculated using the G *Power 3.1 program with a 0.05 significance level, 0.80 power, 0.15 median effect size, and 9 independent variables.

### 2.3. Measures

A structured self-reported questionnaire was used in this study. The questionnaire consisted of a total of 104 items, including 4 items on general characteristics, 6 on job-related characteristics, 6 on the characteristics of the affiliated healthcare institution, 5 on retention intention, and 83 on psychosocial work environment.

#### 2.3.1. Retention Intention

The retention intention, which is the employee’s intention to remain with an organization [22], was measured by the tool developed by Mobley [22] and Becker [23], adapted and modified by Park [24], and supplemented by Lee [25]. The tool consisted of 5 items, which were evaluated on a 5-point scale from “strongly disagree” (1 point) to “strongly agree” (5 points). A higher score indicated greater retention intention. Cronbach’s α was 0.83 in the study by Lee [25], and 0.87 in this study.

#### 2.3.2. Psychosocial Work Environment

The psychosocial work environment was evaluated using the Korean version of the Copenhagen Psychosocial Questionnaire (COPSOQ-K), which Jeon et al. [21] translated after receiving approval. The tool consisted of 7 categories, 27 subcategories, and 83 items. The categories were: demands at work, work organization and job content, collaboration and leadership, work–individual interface, value at the workplace, health and well-being, and aggressive behavior. Each subcategory was evaluated on a 4- or 5-point scale, and the total score was converted to a score out of 100 points. The frequency of aggressive behavior was assessed. For items with positive content, a higher score indicated increased positivity. Cronbach’s α for the COPSOQ ranged between 0.67 and 0.89. Cronbach’s α for the COPSOQ-K ranged between 0.66 and 0.87. For this study, Cronbach’s α was between 0.60 and 0.92.

### 2.4. Data Collection

In the present study, 132 care coordinators participated among 165 nurses in 120 clinics participating in the “Primary Health Care Chronic Diseases Management Pilot Program”. As of July 2020, a total of 1500 clinics participated in the pilot program, and only 120 of them employed nurses. The healthcare institutions’ addresses and representative telephone numbers were collected from the website of the Korean Health Insurance Review and Assessment Service. Recruitment documents detailing the content of the study, including the purpose and methods used, were sent to the institutions. A QR code was attached to the recruitment document to enable those who wanted to participate to register directly and take an online survey. Following this, the purpose and methods used in the study were explained to encourage the recruitment of potential participants in the 120 institutions. Participant recruitment began on 19 October 2020, and data were collected from a total of 132 participants, excluding those who refused to participate in the study, until 19 November 2020.

### 2.5. Data Analysis

Data were analyzed using the Statistical Package for the Social Sciences for Windows Version 26.0 (SPSS Inc., Chicago, IL, USA). Descriptive statistics were conducted to present the general and job-related characteristics of the participants and the characteristics of affiliated health care institutions using frequency, percentage, mean, and standard deviation (SD). Additionally, the retention intentions and psychosocial work environment of the participants were also represented using mean and SD through descriptive statistics. The effects of these characteristics on the retention intentions of participants were analyzed using a hierarchical multiple regression analysis.

### 2.6. Ethical Considerations

This study was approved by the Institutional Review Board (IRB) of E university (IRB No.: 202010-0017-01) and was conducted in compliance with ethical principles for the protection of the participants. The recruitment document included information on the purpose, method, and confidentiality of the study. The participants were also informed that they could withdraw from the study at any time without unfavorable consequences. As it was not possible to interview participants during the COVID-19 pandemic face-to-face, a survey was conducted through an online link after approval of exemption from written consent. The personal information of the participants was coded using a serial number for anonymity. If a participant did not complete the survey, the provided information was not stored. The participants received compensation, and phone numbers that were collected to provide this compensation were immediately discarded.

## 3. Results

### 3.1. General and Job-Related Characteristics of Participants

Table 1 shows the general and job-related characteristics of the participants. All the participants (100%) were women, and their mean age was 45.17 ± 8.20 years. A total of 85.6% of participants were married. Their mean work experience at their current healthcare institution was 8.25 ± 7.25 years. Their mean work experience as care coordinators was 1.41 ± 0.66 years. The average number of patients per day who received face-to-face education and counseling per care coordinator was 7.97 ± 13.79. An average of 5.80 ± 12.82 patients per day underwent telephone counseling with each care coordinator. The average number of doctors, nurses, and nursing assistants per institution was 3.08 ± 4.03, 4.51 ± 6.50, and 4.57 ± 5.64, respectively.

### 3.2. The Effect of Participants’ Psychosocial Work Environment on Their Intention to Stay

Among the psychosocial work environment factors, the organization of work, content of work, and organizational value were found to have an effect on retention intentions. A hierarchical multiple regression analysis was conducted using significant variables of general and job-related characteristics to assess the effects on retention intention. Additionally, psychosocial environment variables were included to evaluate whether the explanatory power increased (see Table 2). Marital status was converted into a dummy variable for analysis. In the regression model, tolerance was greater than 0.1 (0.352–0.787) and the variation inflation factor (VIF) was less than 10 (1.217–2.820), suggesting there were no problems of multicollinearity. The Durbin–Watson coefficient was 1.804, which was close to the reference value of 2. Thus, the independence of residuals was assumed.

There was a significant difference in general and job-related characteristics in stage one. These characteristics included marital status, age, and experience at the current job. Marital status had an effect on retention intention (β = 0.312, *p* = 0.002) with an explanatory power of 9.7% (F = 5.687, *p* = 0.001). In addition to the significantly different general and job-related characteristics, stage two included demands at work, work organization and job content, collaboration and leadership, work–individual interface, value at the workplace, and health and well-being, which are the six categories of psychosocial work environment. As a result, work organization and job content (β = 0.254, *p* = 0.014) and value at the workplace (β = 0.245, *p* = 0.034) had significant effects on retention intention. Work organization and job content, value at the workplace, and marital status were identified as factors influencing the retention intentions of care coordinators, and the final model explained 40.1% of the retention intention (F = 11.830, *p* < 0.001).

## 4. Discussion

This study aimed to investigate the effects of the psychosocial work environment on the retention intentions of chronic disease management care coordinators, and to present basic data to increase the retention rates of care coordinators. Work organization and job content, value at the workplace, and marital status were identified as factors influencing the retention intentions of care coordinators, and the final model explained 40.1% of the retention intention.

Furthermore, among the psychosocial work environment factors, work organization and job content were found to be the most important factors influencing the retention intentions of care coordinators. Work organization and job content are similar to autonomy and are related to the influence of individuals on job content and workload. Our findings are consistent with those of previous studies in which autonomy was determined to have an effect on the job satisfaction and retention intentions of primary care nurses [26,27]. In addition, previous studies showed that patient health management goals were achieved when medication, patient education, and telephone counseling were provided with high autonomy [28,29], which the findings of our study also support. In the United States, Canada, and Germany, professional nurses independently treat patients, conduct physical examinations, and follow up with patients in cooperation with doctors. They provide diagnostic, non-pharmacological, and pharmacological prescriptions, complying with the limits of professional practice for the treatment of patients with chronic disease [5]. In Korea, the scope of a care coordinator’s task and duty is limited, so future studies must seek opportunities for care coordinators to practice their work and determine its scope with autonomy, receive opportunities to develop their competencies, and participate in a more significant manner at work. Some advanced countries permit nurse practitioners to open clinics (Nurse Practitioner-Led Clinics or, NPLCs) to care for patients [30]. However, in Korea, nurse practitioners are not allowed to operate clinics. Article 38 of the Enforcement Rules of the Medical Service Act stipulates the number of nurses in clinic-level medical institutions. However, most clinics can replace nurses with nursing assistants under the “Notice on Nursing Assistant” (Ministry of Health and Welfare Notice No. 90-26).

The present study found value at the workplace—defined in terms of such parameters as trust between executives and colleagues, conflicts, and fair distribution of work—to be another key factor affecting the retention intentions of care coordinators. This finding is in alignment with that of a previous study [31], in which higher expectations of nursing quality on the part of executives led to an improved work environment, and the trust of superiors had a positive effect on achieving the desired organizational goals, thereby acting as an important contributing factor for organizational growth. Therefore, in order to increase the retention intentions of care coordinators, it is fundamental not only to consider personal characteristics such as job autonomy and competency development, but also to develop organizational trust and active improvement measures such as a system of promotion and assignment of new positions.

Work organization and job content were found to have significant effects on the retention intentions of care coordinators. Therefore, further studies must establish the role and function of nurses in primary care by comparing the scope of work, workload, and work environment of nurses and dedicated care coordinators. In addition, care coordinators must receive opportunities to exercise autonomy within their work roles, develop their competencies and participate in a more significant manner at work. Institutional measures to improve the psychosocial work environment must also be evaluated. Moreover, care coordinators must be educated with the latest knowledge of chronic diseases, case analysis, communication techniques, and motivational interview counseling techniques so they can better understand chronic diseases, adapt to clinical settings, and gain recognition for their professionalism and autonomy.

This study has several limitations. First, both care coordinators in charge of the work and care coordinators responsible for other tasks were included in the study, which may not accurately reflect the true characteristics of care coordinators. Furthermore, the COVID-19 pandemic may have decreased the demand for care coordinators, thereby affecting the turnover rate and retention intentions. Changes in the overall healthcare system, such as a decrease in the number of visits of patients with chronic illness and an increase in telephone consultations and demand for chronic disease management, may have been observed as well.

## 5. Conclusions

The psychosocial environment factors that affected the retention intentions of care coordinators were work organization and job content and value at the workplace. Care coordinators must be educated with the latest knowledge of chronic diseases, case analysis, and communication and motivational interview counseling techniques to improve work organization and job content. Furthermore, their professionalism and autonomy must be recognized, as is the case in Canada or the US, where nurses can care for chronic disease patients at nurse practitioner-led clinics. In addition, new policies must be introduced concerning education, counseling, and coordination to improve the professionalism of care coordinators in performing the tasks assigned to them. These measures must be implemented to increase the retention intentions of care coordinators and the efficacy of treatment for patients with chronic illness at primary care institutions.

## Figures and Tables

**Table 1:** General and Job-Related Characteristics of Participants (*N* = 132).

Variables	Categories	*N*	%	M ± SD
Marital status	Married	113	85.6	
	Unmarried	19	14.4	
Age (years)				45.17 ± 8.20
Nursing Education level	Associate’s degree	79	59.8	
	Bachelor’s degree	46	34.8	
	Master’s degree	7	5.3	
Clinical career (years)	Total clinical career			16.16 ± 8.58
	Career in clinic-level healthcare organization			8.25 ± 7.25
	Career as care coordinator			1.41 ± 0.66
Monthly income (KRW 10,000)				249.77 ± 68.08
Employment type	Full time	120	90.9	
	Part time	6	4.5	
	Indefinite term	4	3.0	
	Others	2	1.5	
Number of enrolled patients	<100	55	41.7	
	100–199	22	16.7	
	200–299	35	26.5	
	300	20	15.2	
Number of patients (per day)	Face-to-face			7.97 ± 13.79
	Telehealth			5.80 ± 12.82
Number of employees	Physicians			3.08 ± 4.03
	Nurses			4.51 ± 6.40
	Nurse aides			4.57 ± 5.64

**Table 2:** Factors Influencing Intention to Stay (*N* = 132).

Variables		Model 1			Model 2		
		*β*	t	*p*	*β*	t	*p*
General Characteristics	Marital status	0.312	3.163	0.002	0.228	2.738	0.007
	Age	−0.025	−0.236	0.813	−0.002	−0.025	0.980
Job-Related Characteristics	Career in clinic-level healthcare organization	0.102	1.088	0.279	0.042	0.528	0.598
Psychosocial Work Environment	Demands at work				−0.193	−1.695	0.093
	Work organization and job content				0.254	2.506	0.014
	Collaboration and leadership				0.066	0.621	0.536
	Work–individual interface				0.154	1.797	0.075
	Value at the workplace				0.245	2.149	0.034
	Health and well-being				−0.160	−1.573	0.118
F(*p*)		5.68(<0.001)			11.83(<0.001)		
Adjusted R^2^		0.09			0.40		
R^2^		0.12			0.44		
